# Rpp25 is a major target of autoantibodies to the Th/To complex as measured by a novel chemiluminescent assay

**DOI:** 10.1186/ar4210

**Published:** 2013-04-12

**Authors:** Michael Mahler, Cristina Gascon, Sima Patel, Angela Ceribelli, Marvin J Fritzler, Andreas Swart, Edward KL Chan, Minoru Satoh

**Affiliations:** 1 INOVA Diagnostics, INC. 9900 Old Grove Rd, San Diego, CA 92131-1638, USA; 2 Rheumatology and Clinical Immunology, Humanitas Clinical and Research Center, via Manzoni 56, 20089 Rozzano, and BIOMETRA department, University of Milan, Via Vanvitelli 32, 20129, Milan, Italy; 3 Faculty of Medicine:HRB414, University of Calgary, 3330 Hospital Dr NW, Calgary, AB, T2N 4N1, Canada; 4 Center for Rheumatic Diseases Dr. Gürtler, Kaiser-Friedrich-Str.8, D-41460 Neuss, Germany; 5 Department of Oral Biology, University of Florida, 1395 Center Dr, Gainesville, FL 32610-0424, USA; 6 Division of Rheumatology and Clinical Immunology, Department of Medicine, and Pathology, Immunology and Laboratory Medicine, University of Florida, 1600 SW Archer Rd, Gainesville, FL 32610-0221, USA

## Abstract

**Introduction:**

Autoantibodies to the Th/To antigen have been described in systemic sclerosis (SSc) and several proteins of the macromolecular Th/To complex have been reported to react with anti-Th/To antibodies. However, anti-Th/To has not been clinically utilized due to unavailability of commercial tests. The objective of the present study is to evaluate the newly developed ELISA and chemiluminescent immunoassay (CLIA) to measure autoantibodies to Rpp25 (a component of the Th/To complex) using immunoprecipitation (IP) as the reference method.

**Methods:**

The first cohort consisted of 123 SSc patients including 7 anti-Th/To positive samples confirmed by IP. Additional seven anti-Th/To positive samples from non-SSc patients were also tested. For evaluation of the QUANTA Flash Rpp25 CLIA (research use only), 8 anti-Th/To IP positives, a cohort of 70 unselected SSc patients and sera from various disease controls (*n *= 357) and random healthy individuals (*n *= 10) were studied.

**Results:**

Anti-Rpp25 antibodies determined by ELISA were found in 11/14 anti-Th/To IP positive but only in 1/156 (0.6%) negative samples resulting in a positive percent agreement of 78.6% (95% confidence interval [CI] 49.2, 95.3%) and a negative percent agreement of 99.4% (95% CI 96.4, 100.0%). To verify the results using a second method, 53 samples were tested by ELISA and CLIA for anti-Rpp25 reactivity and the results were highly correlated (rho = 0.71, 95% CI 0.56, 0.81; *P *< 0.0001). To define the cutoff of the CLIA, anti-Th/To IP positive and negative sera were tested using the anti-Rpp25 CLIA. At the cutoff selected by receiver operating characteristic (ROC) analysis 8/8 (100.0%) of the anti-Th/To positive sera but only 2/367 (0.5%) of the controls were positive for anti-Rpp25 antibodies. The positive and negative percent agreements were 100.0% (95% CI 63.1, 100.0%) and 99.5% (95% CI 98.0, 99.9%), respectively. In the disease cohorts 2/70 (2.9%) of the SSc patients were positive for anti-Rpp25 antibodies compared to 2/367 (0.5%) of the controls (*P *= 0.032). ROC analysis showed discrimination between SSc patients and controls with an area under the curve value of 0.732 (95% CI 0.655, 0.809).

**Conclusion:**

Rpp25 is a major target of autoantibodies to the Th/To autoantigen complex. Further studies are needed to evaluate the clinical utility of the new assays.

## Introduction

Systemic autoimmune rheumatic diseases (SARD) including systemic sclerosis (SSc) are characterized by production of autoantibodies to intracellular targets [1]. In SSc, as well as anti-centromere (ACA) [2], anti-topoisomerase I (topo-I, Scl-70) [3] and anti-RNA polymerase III antibodies [1], several other autoantibodies have been described. These include autoantibodies targeting the PM/Scl complex (also known as the exosome) [4], U3RNP/fibrillarin [5] and the Th/To autoantigens [6-9]. Anti-Th/To antibodies are one of the specificities that show homogenous nucleolar staining in indirect immunofluorescence (IIF) antinuclear antibody (ANA) tests [6,10,11]. In SSc, anti-Th/To has been associated with limited cutaneous SSc (lcSSc) subset and the reported prevalence of anti-Th/To antibodies varies between 1 and 13% [6,12,13]. In addition to SSc, a few reports have described anti-Th/To antibodies in rheumatoid arthritis (RA) and interstitial lung disease (ILD) [14,15].

The Th/To antigen complex is a multi-protein-RNA complex (human RNase MRP complex) that consists of a catalytic RNA and several protein components [7,16]. RNase MRP is a ubiquitously expressed eukaryotic endoribonuclease that cleaves various RNAs, including ribosomal, messenger, and mitochondrial RNAs, in a highly specific fashion [7]. At least ten protein subunits, Rpp14, Rpp20, Rpp21, Rpp25, Rpp29 (hPop4) [17], Rpp30 [18], Rpp38 [18], Rpp40, hPop1, and hPop5 are known [7]. Almost all protein components of the RNase MRP and the evolutionarily related RNase P complex have been reported to be the target of autoantibodies in patients with SARD [7,8,14]. The major autoantigens have been identified as Rpp25 and hPop1 [7]. Rpp25 (Ribonuclease P protein subunit p25, NP_060263.2) is a 25 kDa protein subunit of RNase P. Historically, anti-Th/To antibodies have been detected by immunoprecipitation (IP) [6]. While some studies tested serological cohorts, other investigations analyzed samples initially screened based on nucleolar staining pattern identified by IIF. Recently, commercial line immunoassays (LIA) for the detection of anti-Th/To antibodies became available and were evaluated in two independent studies [19,20]. In addition, an IP real-time PCR assay has been developed and evaluated [21].

Although known for over 20 years, little is known about the clinical association of autoantibodies targeting the individual components of the Th/To antigen. Furthermore, anti-Th/To antibodies are rarely used in routine testing algorithms to aid in the diagnosis and stratification of SSc. Consequently, we aimed to develop immunoassays to detect antibodies to a defined single component (Rpp25) of the Th/To complex and to evaluate the newly developed ELISA and chemiluminescent immunoassay (CLIA) using IP as a reference method.

## Methods

### Sera

The first cohort consisted of 123 SSc patients including seven with anti-Th/To positive samples confirmed by IP, enrolled in the University of Florida Center for Autoimmune Diseases (UFCAD) registry from 2000 to 2012. An Additional seven anti-Th/To positive samples from non-SSc patients (two with Raynaud's phenomenon (RP), one with pulmonary hypertension, one with interstitial lung disease (ILD), one with Sjögren's syndrome (SS), one with SS and ILD and one with polymyositis) from the same cohort were also included. For evaluation of QUANTA Flash Rpp25, 53 sera were selected based on the serum volume available. Samples from 70 SSc patients were collected and tested at the University of Calgary (Canada). Sera collected at INOVA included rheumatoid arthritis (RA, *n *= 141), systemic lupus erythematosus (SLE, *n *= 67), undifferentiated connective tissue disease (UCTD, *n *= 17 samples), osteoarthritis (OA, *n *= 47), ankylosing spondylitis (AS, *n *= 13), polymyalgia rheumatica (PMR, *n *= 20), degenerative spine disease (*n *= 6), fibromyalgia (*n *= 5), psoriasis arthritis (*n *= 13), other rheumatological and non-rheumatological diagnoses (*n *= 28) and healthy individuals (HI, *n *= 10). The diagnoses were established as described before [22] or according to the standard disease criteria.

The protocol was approved by the Institutional Review Boards (IRB, Health Research Ethics Board of the University of Calgary and University of Florida). This study meets and is in compliance with all ethical standards in medicine, and informed consent was obtained from all patients according to the Declaration of Helsinki.

#### Recombinant Rpp25 antigen

The cDNA of Rpp25 was cloned into pET28a(+) (Novagen, Merck, Germany) and the his-tagged protein expressed in BL21(DE3) cells according to the manufacturer's instructions. Soluble recombinant Rpp25 was extracted using a detergent and affinity purified using a nickel column. Purity and immunoreactivity was verified by SDS Page and Western blot using anti-HIS antibody (SIGMA # H1029 Monoclonal Anti-poly Histidine antibody, diluted 1:3,000) and a pool of 10 anti-Th/To IP positive sera and one control serum.

### Immunoassays

#### Anti-Rpp25 ELISA

Nunc Immobilizer Amino plates (Thermo Fisher Scientific, Waltham, MA, USA) were coated with 2 microgram/ml of Rpp25 antigen in PBS, 50 μl/well 2 h at 22°C for 2 h and blocked with 0.5% bovine serum albumin (BSA) in NET/NP40 (150 mM NaCl, 2 mM ethylenediaminetetraacetic acid (EDTA), 50 mM Tris-HCl pH 7.5, 0.3% NP40) for 1 h. Wells were then incubated with sera diluted 1:500 in blocking buffer for 1 h at 22°C. After washing three times by TBS/Tween (20 mM Tris-HCl pH 7.5, 150 mM NaCl, 0.1% Tween 20), wells were incubated with alkaline phosphatase-conjugated donkey immunoglobulin (Ig)G F(ab)'2 anti-human IgG (gamma-chain specific, Jackson ImmunoResearch Laboratories, Inc. West Grove, PA, USA) diluted 1:1,000 in blocking buffer for 1 h at 22°C. After washing three times, plates were developed and the OD405 of each sample was converted into units based on the standard curve using the SoftMax Pro 4.7 program (Molecular Devices, Sunnyvale, CA, USA) with four-parameter analysis. The standard curve was established by 1:5 serial dilutions starting from 1:500 dilution of the prototype serum. The last dilution to show optical density (OD) clearly above background was 1:62,500 dilution of the serum, and OD from this dilution was defined as one unit. Units were applied to each dilution as follows, so that the units correlate with the amount of antibodies; 1:500 dilution, 125 units; 1:2,500, 25 units; 1:12,500, 5 units; 1:62,500, 1 unit; 1:312,500, 0.2 units; 1:1,562,500, 0.04 units. Unit values lower than 0.2 units were below the interpretable range of the standard curve and recorded as 0.2 units for data analysis.

#### QUANTA Flash^(R) ^Rpp25

The QUANTA Flash Rpp25 assay (research use only, INOVA Diagnostics Inc., San Diego, CA, USA) is a novel CLIA that is currently used for research purposes only and utilizes the BIO-FLASH^® ^instrument (Biokit s.a., Barcelona, Spain), fitted with a luminometer, as well as all the hardware and liquid handling accessories necessary to fully automate the assay. The principle of the QUANTA Flash assays performed on the BIO-FLASH instrument has recently been described [23].

The QUANTA Flash assay for this study was developed using full-length, purified, recombinant human Rpp25 antigen coated onto paramagnetic beads. Prior to use, the reagent pack containing all the necessary assay reagents is gently inverted thirty times. The sealed reagent tubes are then pierced with the reagent pack lid. A patient's serum sample is pre-diluted with the BIO-FLASH^® ^sample buffer in a small disposable plastic cuvette. Small amounts of the diluted serum, the beads, and the assay buffer are all combined into a second cuvette, mixed, and then incubated for 9.5 minutes at 37°C. The magnetized beads are sedimented using a strong magnet in the washing station and washed several times followed by addition of isoluminol-conjugated anti-human IgG and again incubated 9.5 minutes at 37°C. The magnetized beads are sedimented and washed repeatedly. The isoluminol conjugate is oxidized when sodium hydroxide solution and peroxide solutions (triggers) are added to the cuvette, and the flash of light produced from this reaction is measured as relative light units (RLUs) by the BIO-FLASH^® ^optical system. The RLUs are proportional to the amount of isoluminol conjugate that is bound to the human IgG, which is in turn proportional to the amount of anti-Rpp25 antibodies bound to the antigen on the beads. Precision of the assay and stability of the assay components were verified by precision and stability testing. Total, repeatability, between-run and between-day precision were determined by running the control sample in triplicates, two runs per day over three days resulting in 18 values.

#### Detection of anti-Th/To autoantibodies by immunoprecipitation

Detection of anti-Th/To was based on IP confirmation of the 7-2 and 8-2 RNAs by RNA analysis with urea-PAGE and silver staining (Silver stain plus, Bio-Rad, Hercules, CA, USA). Specificities were verified using previously characterized reference sera. Analysis of proteins to determine other SSc autoantibodies recognized by sera was performed by IP of ^35^S-methionine radiolabeled K562 cell extract, SDS-PAGE and autoradiography as described [6].

### Statistical evaluation

The data were statistically evaluated using the Analyse-it software (Version 1.62; Analyse-it Software, Ltd., Leeds, UK). The chi-square test, Spearman's correlation and Cohen's kappa test of agreement were carried out to analyze the agreement between portions, and *P*-values < 0.05 were considered significant. Receiver-operating characteristics (ROC) analysis was used to analyze the discriminatory ability of different immunoassays.

## Results

### Anti-Rpp25 antibodies in samples characterized by immunoprecipitation and measured by ELISA

**Figure 1:**
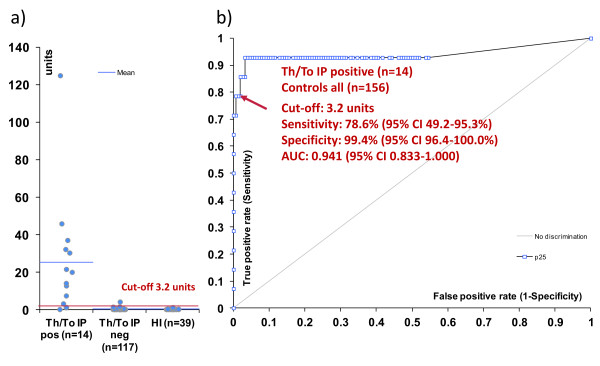
**Anti-Rpp25 antibodies in Th/To immunoprecipitation (IP)-positive and IP-negative samples measured by enzyme-linked immunosorbent assay (ELISA)**. Comparative descriptive analysis is shown in a). In b) the receiver operating characteristic (ROC) analysis shows good discrimination between anti-Th/To IP-positive patients (*n *= 14) and controls (anti-Th/To-negative SSc and healthy individuals, *n *= 156). AUC, area under the curve.

Samples tested for anti-Th/To reactivity by IP were tested for anti-Rpp25 reactivity by ELISA. The reactivity to Rpp25 was significantly higher in anti-Th/To IP-positive than in anti-Th/To IP-negative samples (*P *< 0.0001) (Figure 1). After ROC analysis, showing an area under the curve (AUC) value of 0.941 (95% CI 0.833, 1.000), a preliminary cutoff was defined, which yielded a sensitivity of 78.6% (95% CI 49.2, 95.3%) and a specificity of 99.4% (95% CI 96.4, 100.0%). There was no significant difference in the levels of anti-Rpp25 between anti-Th/To-positive SSc vs. anti-Th/To-positive non-SSc patients (*P *= 0.902).

### Anti-Rpp25 antibodies measured by chemiluminescent technology

**Figure 2:**
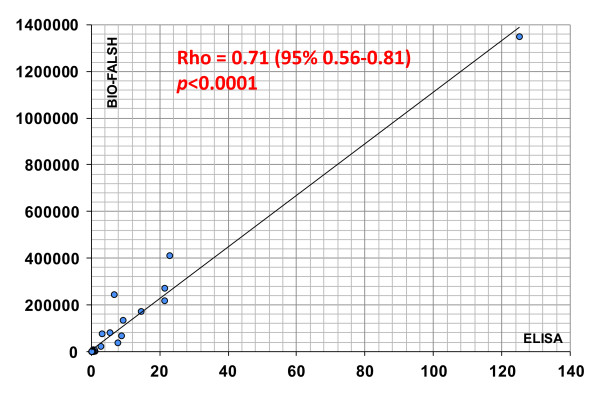
**Correlation between enzyme-linked immunosorbent assay (ELISA) and chemiluminescent immunoassay (CLIA)**. The results of 53 samples tested by anti-Rpp25 ELISA and CLIA (QUANTA Flash) showed good agreement (*rho *= 0.71; *P *< 0.0001, Spearman correlation test).

**Figure 3:**
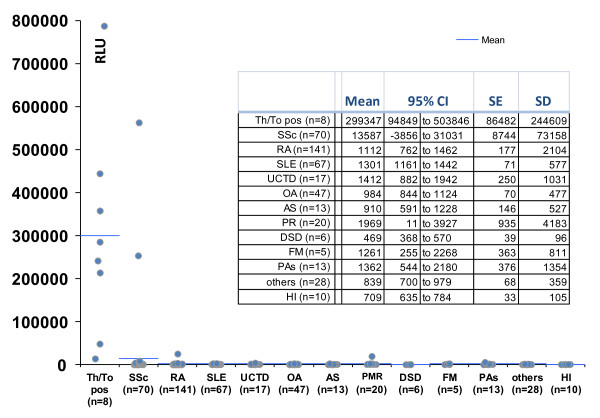
**Anti-Rpp25 antibodies in samples from anti-Th/To immunoprecipitation (IP)-positive patients, an independent cohort of patients with systemic sclerosis and controls measured by QUANTA Flash anti-Rpp25**. Comparative descriptive analysis is shown of samples from anti-Th/To IP-positive patients (*n *= 8), patients with SSc (*n *= 70) and controls (*n *= 367) including patients with rheumatoid arthritis (RA, *n *= 141), systemic lupus erythematosus (SLE, *n *= 67), undifferentiated connective tissue disease (UCTD, *n *= 17), osteoarthritis (OA, *n *= 47), ankylosing spondylitis (AS, *n *= 13), polymyalgia rheumatica (PR, *n *= 20), degenerative spine disease (*n *= 6), fibromyalgia (*n *= 5), psoriasis arthritis (*n *= 13), other pathologies (*n *= 28) and healthy individuals (HI, *n *= 10). SE, standard error.

**Figure 4:**
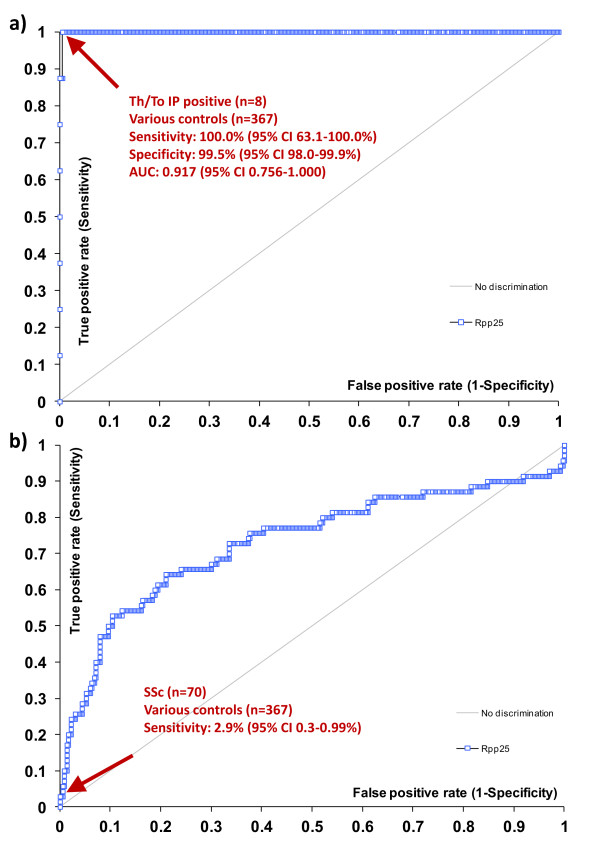
**Receiver-operating characteristics (ROC) analysis**. a) ROC analysis shows good discrimination between Th/To immunoprecipitation (IP)-positive patients (*n *= 8) and controls (*n *= 367) including rheumatoid arthritis (RA, *n *= 141), systemic lupus erythematosus (SLE, *n *= 67), undifferentiated connective tissue disease (UCTD, *n *= 17), osteoarthritis (OA, *n *= 47), ankylosing spondylitis (AS, *n *= 13), polymyalgia rheumatica (PMR, *n *= 20), degenerative spine disease (*n *= 6), fibromyalgia (*n *= 5), psoriasis arthritis (*n *= 13), other pathologies (*n *= 28) and healthy individuals (HI, *n *= 10). b) ROC analysis shows discrimination between SSc (*n *= 70) and controls (*n *= 367). AUC, area under the curve; SSc, systemic sclerosis.

To verify the results using a second method, a total of 53 samples that were tested by ELISA and QUANTA Flash CLIA for anti-Rpp25 reactivity showed high correlation (*rho *= 0.71, 95% CI 0.56, 0.81; *P *< 0.0001; see Figure 2). Next we analyzed the precision of the QUANTA Flash Rpp25 CLIA, which demonstrated good precision with a total variation of 6.6% (Table 1). To analyze the prevalence of anti-Rpp25 antibodies in different cohorts, anti-Th/To-positive sera (*n *= 8 identified by IP), unselected SSc samples (*n *= 70) and disease and healthy controls (*n *= 367) were tested using the anti-Rpp25 assay on the BIO-FLASH instrument (Figure 3). The anti-Th/To-positive sera were from four SSc patients, two with RP, one with SS and one with ILD. There was no significant difference between the Th/To-positive SSc and non-SSc patients (*P *= 0.4857). When comparing anti-Th/To IP-positive samples and controls by ROC analysis, showing an AUC value of 0.919 (95% CI 0.919, 1.000), a preliminary cutoff was defined (10,000 RLU, see Figure 4). At this cutoff 8/8 (100.0%) of the anti-Th/To-positive sera but only 2/367 (0.5%) of the controls were positive for anti-Rpp25 antibodies by QUANTA Flash Rpp25 (*P *< 0.0001). Thus a positive percent agreement of 100.0% (95% CI 63.1, 100.0%) and a negative percent agreement of 99.5% (95% CI 98.0, 99.9%) were found. At this cutoff, 2/70 (2.9%) of the second cohort of SSc patients were positive for anti-Rpp25 antibodies compared to 2/367 (0.5%) of the controls (*P *= 0.032). Positive (LR+) and negative (LR-) likelihood ratios were 5.24 and 0.98, respectively (Table 2). The two control patients with a positive result had low levels of anti-Rpp25 antibodies and one had RA (RLU = 25,259) and the other patient had a diagnosis of PR (RLU = 19,561). ROC analysis showed discrimination between SSc patients and controls with AUC values of 0.732 (95% CI 0.655, 0.809).

**Table 1:** Precision of QUANTA Flash for the detection of anti-Th/To (Rpp25) antibodies

Precision	SD (95% CI)	Coefficient of variation	Claimed SD	*P*
Total	1,665.1 (1124.7-3189.9)	6.6%	3,786.1	0.9919
Repeatability	1,452.0 (1041.2-2396.9)	5.8%	3,786.1	0.9997
Between-run	0.0	0.0%		
Between-day	814.9	3.2%		

**Table 2:** Clinical sensitivity and specificity of anti-Th/To antibodies measured by QUANTA Flash Rpp25 vs a commercial line immunoassay for systemic sclerosis (SSc)

	**Villalta *et al***.	**Bonroy *et al***.	Present study
Method	EUROLINE SSc Profile	EUROLINE SSc Profile	QUANTA Flash Rpp25 (CLIA)
Sensitivity^1^	7/210 (3.3; 1.3, 6.7)	3/145 (2.1; 0.4, 5.9)	2/70 (2.9; 0.3, 9.9)
Specificity^2^	148/150 (98.7; 95.2, 99.9)	271/277 (97.8; 95.3, 99.2)	365/367 (99.5; 98.0, 99.9)
LR+	2.5	0.96	5.24
LR-	0.98	1.0	0.98

^1^Values are presented as number of positives/number of SSc samples tested (percent positive; 95% CI). ^2^Values are presented as number of negatives/number of non-SSc samples tested (percent negative; 95%CI). LR+, positive likelihood ratio; LR-, negative likelihood ratio; CLIA, chemiluminescent assay.

## Discussion

Autoantibodies represent valuable biomarkers in the diagnosis of SARD, including SSc [1,24]. Almost all protein components of the RNase MRP and the evolutionarily related RNase P complex have been reported to be the target of autoantibodies in patients with SARD [7,14]. In our study we focused on the autoantibody reactivity to Rpp25, one of the major antigens of the complex [7].

Our data show that Rpp25 is a major autoantigen targeted by anti-Th/To antibodies, which is consistent with previous findings [7]. In our cohort of samples, anti-Rpp25 was detected in approximately 70 to100% (depending on the assay and cutoff used) of anti-Th/To reactivity. In the study by van Eenennaam *et al. *[7], 8/12 (66.7%) anti-Th/To-positive samples immunoprecipitated *in-vitro *transcribed and translated Rpp25. Both assays used in our study (ELISA and CLIA) showed good discrimination between anti-Th/To IP-positive and IP-negative samples and exhibited excellent correlation with each other. However, the CLIA showed a superior discrimination (signal to noise ratio) between anti-Th/To-negative and -positive samples and might represent the preferred method to detect antibodies to Rpp25.

The high sensitivity of the BIO-FLASH CLIA system is interesting. There are several technical differences between the ELISA and the CLIA which might account for the better discrimination between anti-Th/To-positive and -negative samples. The BIO-FLASH system uses magnetic particles that have a significantly higher surface area compared to ELISA plates. On average, the CLIA uses about 5 to10 times more antigen per reaction compared to ELISA (unpublished data). In addition, the antibody reaction occurs in solution and follows microfluidic kinetics [25]. The samples from the anti-Th/To-positive RA and PMR patients did not show a nucleolar staining pattern. Although they were not tested by IP for anti-Th/To reactivity, it is likely that they represent false positive findings. Despite those two samples, the specificity of the Rpp25 CLIA is very high (> 99%). Although the reason for the difference between the anti-Rpp25 CLIA and ELISA remains speculative and deserves further investigation, the anti-Th/To (Rpp25) CLIA holds promise to provide a reliable alternative method to the radioisotope-based IP assay.

Although anti-Th/To antibodies are uncommon in samples from patients with SARD, the observation that anti-Th/To antibodies are mostly detectable in SSc makes this specificity important in the diagnosis of SSc. In addition, current multiplex assays [26] and a screening fluorescence enzyme immunoassay (FEIA) [27] show satisfactory performance characteristics as an ANA screening test for mixed connective tissue disease (MCTD), SS and autoimmune myositis (AIM), but are not sufficiently sensitive for SSc due to the lack of nucleolar antigens [28]. An anti-Th/To test may also be applicable to non-SSc patients such as those with ILD, since anti-Th/To antibodies have been reported in around 50% of patients with anti-nucleolar antibody-positive idiopathic pulmonary fibrosis [10]. The prevalence in our unselected cohort of SSc patients was 2.9%, similar to recent studies (Table 2). Villalta [19] and Bonroy [20] found anti-Th/To antibodies in 3.3% and 2.1% of SSc patients, respectively, but observed lower specificity (98.7% and 97.8% vs 99.5% in our study).

In a study by Kuwana *et al. *[14], anti-hPop1 antibodies were significantly more prevalent in anti-Th/To-positive SSc patients, compared to anti-Th/To-positive patients with other types of SARD. In contrast, Rpp30 and Rpp38 were equally targeted by antibodies from SSc and non-SSc SARD patients. Further studies with additional Th/To recombinant or purified proteins are required to verify this finding.

Although known for over 20 years, understanding the clinical features associated with the anti-Th/To system requires clarification. Previous studies are mostly consistent in showing its association with lcSSc; however, association with more specific clinical features are somewhat inconsistent. A small number of anti-Th/To positive patients, differences in ethnicity and environment, recruitment bias and other types of bias could explain the inconsistency [6,8,29-31]. Anti-Th/To antibodies have been associated with pericarditis and ILD, and they are highly frequent in intrinsic pulmonary hypertension [6,12]. Compared with the ACA patients, anti-Th/To lcSSc patients have more subtle cutaneous, vascular, and gastrointestinal involvement, but more often have certain features typically seen in diffuse scleroderma, such as pulmonary fibrosis and scleroderma renal crisis, as well as reduced survival compared to ACA-positive patients [29]. Like other SSc-related autoantibodies, in patients with Raynaud's phenomenon anti-Th/To antibodies are risk factors that are predictive of emerging SSc [32]. Anti-Th/To-positive patients demonstrate earlier development of abnormalities on nail-fold capillary microscopy (NCM) than ACA-positive patients [32]. There is some evidence that anti-Th/To-positive patients are younger and more frequently male compared to ACA positive patients [6], and it has been reported that the prevalence of anti-Th/To antibodies might be higher in Caucasian Americans compared to African and Latin Americans [30]. Although we did not analyze associations between anti-Rpp25 antibodies and clinical features in our SARD cohort, it is likely that associations of anti-Rpp25 and anti-Th/To are similar based on the good correlation between anti-Rpp25 antibodies (by ELISA or CLIA) and anti-Th/To antibodies (by IP).

Although the prevalence of anti-Th/To antibodies is relatively low, testing for those antibodies might have significant value for patient stratification [5,33]. In a previous study dcSSc and lcSSc subsets were associated with particular organ manifestations, but in this analysis the clinical distinction appeared superseded by an antibody-based classification in predicting some SSc-related complications [33]. In addition, the discrimination between SSc and controls as measured by the AUC (0.73) derived from ROC analysis using anti-Th/To (Rpp25) antibody results was similar to the AUC (0.67) generated by ACA [34].

Further studies using large cohorts of SSc patients such as those collected by EULAR Scleroderma Trials and Research (EUSTAR) [35], the Canadian Scleroderma Research Group (CSRG) [36] or the German Network for systemic scleroderma [13] and the Australian cohort [37] are needed to analyze the clinical utility of antibodies to Rpp25.

## Conclusions

Rpp25 is a major target of autoantibodies to the Th/To autoantigen complex. Autoantibodies to Rpp25 detected by ELISA and especially CLIA show excellent agreement with anti-Th/To antibodies detected by IP. The CLIA represents a quick and reliable method that allows the detection of anti-Th/To antibodies in as little as 30 minutes. Further studies are needed to evaluate the clinical utility of the new assays.

## Abbreviations

ACA: anti-centromere antibodies; AIM: autoimmune myositis; ANA: antinuclear antibodies; AS: ankylosing spondylitis; AUC: area under the curve; BSA: bovine serum albumin; CLIA: chemiluminescent immunoassay; ELISA: enzyme-linked immunosorbent assay; FEIA: fluorescence enzyme immunoassay; HI, healthy individuals; IIF: indirect immunofluorescence; ILD: interstitial lung disease; IP: immunoprecipitation; lcSSc: limited cutaneous systemic sclerosis; LIA: line immunoassay; MCTD: mixed connective tissue disease; NCM: nail-fold capillary microscopy; OA: osteoarthritis; PBS: phosphate-buffered saline; PCR; polymerase chain reaction; PM: polymyositis; PMR: polymyalgia rheumatica; RA: rheumatoid arthritis; RLU: relative light units; ROC: receiver-operating characteristics; RP: Raynaud's phenomenon; SARD: systemic autoimmune rheumatic disease; SS: Sjögren's syndrome; SLE: systemic lupus erythematosus; SSc: systemic sclerosis; UCTD: undifferentiated connective tissue disease; UFCAD: University of Florida Center for Autoimmune Diseases.

## Competing interests

M Mahler, C Gascon and S Patel are employed at INOVA diagnostics selling autoantibody assays. M Fritzler is a consultant to INOVA Diagnostics, ImmunoConcepts Inc. and has received gifts in kind from Euroimmun GmbH. The other authors have no conflict of interest.

## Authors' contributions

MM and MS designed the study, had full access to all of the data in the study, analyzed the data, take responsibility for the integrity of the data and the accuracy of the data analysis, interpreted the data, and drafted the manuscript. EKLC helped to design the study and contributed to the overall project management. MJF, AS and AC enrolled patients for the study and contributed to the data. CG developed and optimized the recombinant antigen and developed the bead coupling chemistry. SP developed the bead coupling chemistry, tested samples and analyzed data. MM interpreted the data and drafted the manuscript, was responsible for overall project management and designed the study. All authors critically revised the manuscript and read and approved the final manuscript for publication.

## References

[B1] Mahler M Fritzler MJ Epitope specificity and significance in systemic autoimmune diseases Ann N Y Acad Sci 2010 1183 267 287 10.1111/j.1749-6632.2009.05127.x 20146721

[B2] Fritzler MJ Rattner JB Luft LM Edworthy SM Casiano CA Peebles C Mahler M Historical perspectives on the discovery and elucidation of autoantibodies to centromere proteins (CENP) and the emerging importance of antibodies to CENP-F Autoimmun Rev 2011 10 194 200 10.1016/j.autrev.2010.09.025 20933614

[B3] Mahler M Silverman ED Schulte-Pelkum J Fritzler MJ Anti-Scl-70 (topo-I) antibodies in SLE: Myth or reality? Autoimmun Rev 2010 9 756 760 10.1016/j.autrev.2010.06.005 20601198

[B4] Mahler M Raijmakers R Novel aspects of autoantibodies to the PM/Scl complex: clinical, genetic and diagnostic insights Autoimmun Rev 2007 6 432 437 10.1016/j.autrev.2007.01.013 17643929

[B5] Steen VD Autoantibodies in systemic sclerosis Semin Arthritis Rheum 2005 35 35 42 10.1016/j.semarthrit.2005.03.005 16084222

[B6] Ceribelli A Cavazzana I Franceschini F Airo P Tincani A Cattaneo R Pauley BA Chan EK Satoh M Anti-Th/To are common antinucleolar autoantibodies in Italian patients with scleroderma J Rheumatol 2010 37 2071 2075 10.3899/jrheum.100316 20682663

[B7] Van Eenennaam H Vogelzangs JH Lugtenberg D Van Den Hoogen FH Van Venrooij WJ Pruijn GJ Identity of the RNase MRP- and RNase P-associated Th/To autoantigen Arthritis Rheum 2002 46 3266 3272 10.1002/art.10673 12483731

[B8] Van Eenennaam H Vogelzangs JH Bisschops L Te Boome LC Seelig HP Renz M De Rooij DJ Brouwer R Pluk H Pruijn GJ Van Venrooij WJ Van Den Hoogen FH Autoantibodies against small nucleolar ribonucleoprotein complexes and their clinical associations Clin Exp Immunol 2002 130 532 540 10.1046/j.1365-2249.2002.01991.x 12452846 PMC1906554

[B9] Okano Y Medsger TA Jr Autoantibody to Th ribonucleoprotein (nucleolar 7-2 RNA protein particle) in patients with systemic sclerosis Arthritis Rheum 1990 33 1822 1828 10.1002/art.1780331210 1701994

[B10] Fischer A Pfalzgraf FJ Feghali-Bostwick CA Wright TM Curran-Everett D West SG Brown KK Anti-th/to-positivity in a cohort of patients with idiopathic pulmonary fibrosis J Rheumatol 2006 33 1600 1605 16783860

[B11] Wiik AS Hoier-Madsen M Forslid J Charles P Meyrowitsch J Antinuclear antibodies: a contemporary nomenclature using HEp-2 cells J Autoimmun 2010 35 276 290 10.1016/j.jaut.2010.06.019 20650611

[B12] Graf SW Hakendorf P Lester S Patterson K Walker JG Smith MD Ahern MJ Roberts-Thomson PJ South Australian Scleroderma Register: autoantibodies as predictive biomarkers of phenotype and outcome Int J Rheum Dis 2012 15 102 109 10.1111/j.1756-185X.2011.01688.x 22324953

[B13] Mierau R Moinzadeh P Riemekasten G Melchers I Meurer M Reichenberger F Buslau M Worm M Blank N Hein R Muller-Ladner U Kuhn A Sunderkotter C Juche A Pfeiffer C Fiehn C Sticherling M Lehmann P Stadler R Schulze-Lohoff E Seitz C Foeldvari I Krieg T Genth E Hunzelmann N Frequency of disease-associated and other nuclear autoantibodies in patients of the German Network for Systemic Scleroderma: correlation with characteristic clinical features Arthritis Res Ther 2011 13 R172 10.1186/ar3495 22018289 PMC3308107

[B14] Kuwana M Kimura K Hirakata M Kawakami Y Ikeda Y Differences in autoantibody response to Th/To between systemic sclerosis and other autoimmune diseases Ann Rheum Dis 2002 61 842 846 10.1136/ard.61.9.842 12176814 PMC1754228

[B15] Koenig M Fritzler MJ Targoff IN Troyanov Y Senecal JL Heterogeneity of autoantibodies in 100 patients with autoimmune myositis: insights into clinical features and outcomes Arthritis Res Ther 2007 9 R78 10.1186/ar2276 17688695 PMC2206383

[B16] Mehra S Walker J Patterson K Fritzler MJ Autoantibodies in systemic sclerosis Autoimmun Rev 2013 12 340 354 10.1016/j.autrev.2012.05.011 22743034

[B17] Guerrier-Takada C Eder PS Gopalan V Altman S Purification and characterization of Rpp25, an RNA-binding protein subunit of human ribonuclease P RNA 2002 8 290 295 10.1017/S1355838202027954 12003489 PMC1370251

[B18] Eder PS Kekuda R Stolc V Altman S Characterization of two scleroderma autoimmune antigens that copurify with human ribonuclease P Proc Natl Acad Sci USA 1997 94 1101 1106 10.1073/pnas.94.4.1101 9037013 PMC19751

[B19] Villalta D Imbastaro T Di Giovanni S Lauriti C Gabini M Turi MC Bizzaro N Diagnostic accuracy and predictive value of extended autoantibody profile in systemic sclerosis Autoimmun Rev 2012 12 114 120 10.1016/j.autrev.2012.07.005 22776784

[B20] Bonroy C Van Praet J Smith V Van Steendam K Mimori T Deschepper E Deforce D Devreese K De Keyser F Optimization and diagnostic performance of a single multiparameter lineblot in the serological workup of systemic sclerosis J Immunol Methods 2012 379 53 60 10.1016/j.jim.2012.03.001 22446156

[B21] Ceribelli A Satoh M Chan EK A new immunoprecipitation-real time quantitative PCR assay for anti-Th/To and anti-U3RNP antibody detection in systemic sclerosis Arthritis Res Ther 2012 14 R128 10.1186/ar3858 22643159 PMC3446509

[B22] Mahler M Fritzler MJ Bluthner M Identification of a SmD3 epitope with a single symmetrical dimethylation of an arginine residue as a specific target of a subpopulation of anti-Sm antibodies Arthritis Res Ther 2005 7 R19 R29 10.1186/ar1455 15642139 PMC1064884

[B23] Mahler M Radice A Yang W Bentow C Seaman A Bianchi L Sinico RA Development and performance evaluation of novel chemiluminescence assays for detection of anti-PR3 and anti-MPO antibodies Clin Chim Acta 2012 413 719 726 10.1016/j.cca.2012.01.004 22265712

[B24] Satoh M Vazquez-Del Mercado M Chan EK Clinical interpretation of antinuclear antibody tests in systemic rheumatic diseases Mod Rheumatol 2009 19 219 228 10.1007/s10165-009-0155-3 19277826 PMC2876095

[B25] Thompson JA Bau HH Microfluidic, bead-based assay: Theory and experiments J Chromatogr B Analyt Technol Biomed Life Sci 2010 878 228 236 10.1016/j.jchromb.2009.08.050 19766545 PMC2818129

[B26] Op De Beeck K Vermeersch P Verschueren P Westhovens R Marien G Blockmans D Bossuyt X Antinuclear antibody detection by automated multiplex immunoassay in untreated patients at the time of diagnosis Autoimmun Rev 2012 12 137 143 10.1016/j.autrev.2012.02.013 22387973

[B27] Parker JC Bunn CC Sensitivity of the Phadia EliA connective tissue disease screen for less common disease-specific autoantibodies J Clin Pathol 2011 64 631 633 10.1136/jcp.2010.084756 21220786

[B28] Shanmugam VK Swistowski DR Saddic N Wang H Steen VD Comparison of indirect immunofluorescence and multiplex antinuclear antibody screening in systemic sclerosis Clin Rheumatol 2011 30 1363 1368 10.1007/s10067-011-1766-6 21614475 PMC3664239

[B29] Mitri GM Lucas M Fertig N Steen VD Medsger TA Jr A comparison between anti-Th/To- and anticentromere antibody-positive systemic sclerosis patients with limited cutaneous involvement Arthritis Rheum 2003 48 203 209 10.1002/art.10760 12528120

[B30] Krzyszczak ME Li Y Ross SJ Ceribelli A Chan EK Bubb MR Sobel ES Reeves WH Satoh M Gender and ethnicity differences in the prevalence of scleroderma-related autoantibodies Clin Rheumatol 2011 30 1333 1339 10.1007/s10067-011-1751-0 21523365 PMC3629275

[B31] Walker JG Fritzler MJ Update on autoantibodies in systemic sclerosis Curr Opin Rheumatol 2007 19 580 591 10.1097/BOR.0b013e3282e7d8f9 17917539

[B32] Koenig M Joyal F Fritzler MJ Roussin A Abrahamowicz M Boire G Goulet JR Rich E Grodzicky T Raymond Y Senecal JL Autoantibodies and microvascular damage are independent predictive factors for the progression of Raynaud's phenomenon to systemic sclerosis: a twenty-year prospective study of 586 patients, with validation of proposed criteria for early systemic sclerosis Arthritis Rheum 2008 58 3902 3912 10.1002/art.24038 19035499

[B33] Walker UA Tyndall A Czirjak L Denton C Farge-Bancel D Kowal-Bielecka O Muller-Ladner U Bocelli-Tyndall C Matucci-Cerinic M Clinical risk assessment of organ manifestations in systemic sclerosis: a report from the EULAR Scleroderma Trials And Research group database Ann Rheum Dis 2007 66 754 763 10.1136/ard.2006.062901 17234652 PMC1954657

[B34] Mahler M Maes L Blockmans D Westhovens R Bossuyt X Riemekasten G Schneider S Hiepe F Swart A Gurtler I Egerer K Fooke M Fritzler MJ Clinical and serological evaluation of a novel CENP-A peptide based ELISA Arthritis Res Ther 2010 12 R99 10.1186/ar3029 20487535 PMC2911886

[B35] Meier FM Frommer KW Dinser R Walker UA Czirjak L Denton CP Allanore Y Distler O Riemekasten G Valentini G Muller-Ladner U Update on the profile of the EUSTAR cohort: an analysis of the EULAR Scleroderma Trials and Research group database Ann Rheum Dis 2012 71 1355 1360 10.1136/annrheumdis-2011-200742 22615460

[B36] Hudson M Pope J Mahler M Tatibouet S Steele R Baron M Fritzler MJ Clinical significance of antibodies to Ro52/TRIM21 in systemic sclerosis Arthritis Res Ther 2012 14 R50 10.1186/ar3763 22394602 PMC3446416

[B37] Nikpour M Hissaria P Byron J Sahhar J Micallef M Paspaliaris W Roddy J Nash P Sturgess A Proudman S Stevens W Prevalence, correlates and clinical usefulness of antibodies to RNA polymerase III in systemic sclerosis: a cross-sectional analysis of data from an Australian cohort Arthritis Res Ther 2011 13 R211 10.1186/ar3544 22189167 PMC3334664

